# Intratumoral immunoglobulin isotypes predict survival in lung adenocarcinoma subtypes

**DOI:** 10.1186/s40425-019-0747-1

**Published:** 2019-10-29

**Authors:** O. I. Isaeva, G. V. Sharonov, E. O. Serebrovskaya, M. A. Turchaninova, A. R. Zaretsky, M. Shugay, D. M. Chudakov

**Affiliations:** 1 Center of Life Sciences, Skolkovo Institute of Science and Technology, Moscow, Russia; 2 BostonGene LLC, Lincoln, MA USA; 3 Laboratory of Genomics of Antitumor Adaptive Immunity, Privolzhsky Research Medical University, Nizhny Novgorod, Russia; 4 Genomics of Adaptive Immunity Department, Shemyakin and Ovchinnikov Institute of Bioorganic Chemistry, Moscow, Russia; 5 Institute of Translational Medicine, Pirogov Russian National Research Medical University, Moscow, Russia; 6 Evrogen JSC, Moscow, Russia

## Abstract

**Background:**

The role of tumor-infiltrating B-cells (TIBs) and intratumorally-produced antibodies in cancer-immunity interactions essentially remains *terra incognita*. In particular, it remains unexplored how driver mutations could be associated with distinct TIBs signatures and their role in tumor microenvironment.

**Methods:**

Here we analyzed associations of immunoglobulin isotypes and clonality with survival in TCGA RNA-Seq data for lung adenocarcinoma (LUAD), stratifying patients into 12 driver mutation and phenotypic tumor subgroups.

**Results:**

We revealed several unexpected associations between TIBs behavior and prognosis. Abundance and high proportion of IgG1 isotype, and low proportion of IgA among all intratumorally produced immunoglobulins were specifically associated with improved overall survival for *KRAS*^mut^ but not *KRAS*^wt^ LUAD, revealing the first link between a driver mutation and B-cell response. We found specific IgG1 signature associated with long survival, which suggests that particular specificities of IgG1+ TIBs could be beneficial in *KRAS*^mut^ LUAD. In contrast to our previous observations for melanoma, highly clonal IgG1 production by plasma cells had no meaningful effect on prognosis, suggesting that IgG1+ TIBs may exert a beneficial effect in *KRAS*^mut^ cases in an alternative way, such as efficient presentation of cognate antigens or direct B cell attack on tumor cells. Notably, a high proportion of the IgG1 isotype is positively correlated with the non-silent mutation burden both in the general LUAD cohort and in most patient subgroups, supporting a role for IgG1^+^ TIBs in antigen presentation. Complementing the recent finding that the presence of stromal IgG4-producing cells is associated with a favorable prognosis for patients with stage I squamous cell carcinoma, we show that the abundance of IgG4-producing TIBs likewise has a strong positive effect on overall survival in *STK11*^mut^ and proximal proliferative subgroups of LUAD patients. We hypothesize that the positive role of IgG4 antibodies in some of the lung cancer subtypes could be associated with reported inability of IgG4 isotype to form immune complexes, thus preventing immunosuppression via activation of the myeloid-derived suppressor cell (MDSC) phenotype.

**Conclusions:**

We discover prominent and distinct associations between TIBs antibody isotypes and survival in lung adenocarcinoma carrying specific driver mutations. These findings indicate that particular types of tumor-immunity relations could be beneficial in particular driver mutation context, which should be taken into account in developing strategies of cancer immunotherapy and combination therapies. Specificity of protective B cell populations in specific cancer subgroups could become a clue to efficient targeted immunotherapies for appropriate cohorts of patients.

## Background

Recent work has revealed the importance of the antigenic specificity [1–3], clonality [4, 5], hypermutation [5, 6], and isotype [5–8] of TIBs, circulating plasmablasts [9], and serum self- and tumor-specific antibodies [10, 11] in tumor-immunity interactions. In particular, TIBs and tumor-infiltrating plasma cells—or more accurately, intra-tumoral B cells, since these may also be generated in local tertiary lymphoid structures (TLS) [1, 12–15]—are believed to play important roles in the tumor microenvironment. Their major modes of action include presentation of B-cell receptor (BCR)-cognate antigens to CD4^+^ and potentially CD8^+^ T-cells [15–18], production of cytokines that can stimulate or suppress anti-tumor response [19–21], and production of tumor-specific antibodies [1–3] that may enhance killing of tumor cells via ADCC [8, 22], enhance antigen capture and presentation by dendritic cells [2], or form immune complexes that promote activation of MDSCs [23, 24].

The involvement of specific BCRs and antibodies in both antitumor—and potentially, pro-tumor—reactions is becoming increasingly clear. Serum auto-antibodies against tumor-associated and self-antigens have been validated as biomarkers for the early detection of cancer [11, 25, 26] and could also serve as useful prognostic markers at later stages of disease [27, 28]. Blood plasmablast counts and BCR clonality were recently shown to be hallmarks of non-progressing cancer in patients treated with anti-CTLA4 immunotherapy, and the therapy itself led to an increase in these parameters [9]. The amount of B-cells and plasma cells in a tumor is also associated with a good prognosis for diverse solid tumors [29–32]. On the other hand, several clinical studies of hepatocellular carcinoma [33], prostate [21], renal [34], and breast [35] cancer have indicated that high B-cell or plasma cell content may be associated with a negative prognosis. This highlights the possibility that B cells can also help maintain an immunosuppressive microenvironment, a function that is sometimes attributed to a certain population of B-cells referred as regulatory B-cells [36, 37].

Antibody functionality is strongly influenced by isotype [38], and this feature is likely to be an important piece of the puzzle in terms of understanding B-cell-tumor interactions. Particular isotypes could also be associated with specific B-cell functionalities. For example, IgA+ plasma cells have been described as preferentially producing the immunosuppressive IL-10 and PD-L1 in some cancers [21, 33].

Based on our analysis of RNA-Seq data from TCGA, we recently demonstrated that high expression levels, proportion, and clonality (i.e., focused expression of particular clonal variants) of cytotoxic IgG1 antibodies is associated with a markedly better prognosis in melanoma, while a high proportion of the IgA isotype is associated with shorter survival [39].

Here, we have investigated the role of different antibody isotypes and clonality in lung adenocarcinoma (LUAD), splitting the 442 patients available in the TCGA database into relevant subgroups based on the presence of key driver mutations or transcriptional subtypes. We reveal previously unexpected associations between dominating isotypes of TIBs and survival in specific subgroups of LUAD patients. Our results for the first time link driver mutations and B cell response in tumor microenvironment, and suggest that patient stratification for immunotherapies and design of combination therapies should take both these parameters in consideration.

## Methods

### Initial data filtering

Patient data from the TCGA LUAD project was obtained from the GDC portal repository (https://portal.gdc.cancer.gov/). We downloaded the HTSeq-FPKM files and transformed the transcript-level data into gene-level data by summarizing alternative transcripts. FPKM were then transformed to TPM. Samples from formalin-fixed paraffin-embedded (FFPE) tissue, normal tissue or metastatic lesions as well as entities with warnings were removed. One sample for each patient was selected in accordance with GDC recommendations (https://confluence.broadinstitute.org/display/GDAC/FAQ#FAQ-replicateFilteringQ%C2%A0Whatdoyoudowhenmultiplealiquotbarcodesexistforagivensampleportionanalytecombination) After such filtration, a cohort of 442 patients was formed (“general cohort” in our study).

### Mutation information

Mutation information was also obtained from GDC portal. Mutations with low Variant Effect Predictor (VEP) impact that were not annotated by SIFT or PolyPhen as having impact were excluded from analysis. We identified the following numbers of patients with relevant genotypes: *KRAS*^mut^, 122 patients; *KRAS*^wt^, 320 patients; *STK11*^mut^, 73 patients; *STK11*^wt^, 369 patients; *TP53*^mut^, 220 patients; *TP53*^wt^, 222 patients; *EGFR*^mut^, 57 patients.

### PD-L1 levels

PD-L1 levels were characterized based on *CD274* gene expression data. Samples that had *CD274* expression higher than twice the mean value in the general cohort were assigned to the PD-L1^high^ group (*N* = 51); the remaining samples formed the PD-L1^low^ group (*N* = 391).

### IGH, IGA and IGHG

Total BCR/antibody expression (IGH) was calculated as a sum of the expressions of *IGHA1, IGHA2, IGHG1, IGHG2, IGHG3, IGHG4, IGHM, IGHD* and *IGHE* genes. IgA expression was calculated as a sum of expression values for the *IGHA1* and *IGHA2* genes.

### IGHV-IGHJ signature

Principal component analysis (prcomp function in R) was used to determine IGHV-IGHJ profiles that explain the most variance across the LUAD cohort. This analysis was limited to IgG1 CDR3 clonotypes, samples with less than 500 IgG1 CDR3-covering reads were removed. High and low survival was determined by comparing to median survival in corresponding cohort. Note that we limited to IGHV families, so e.g. IGHV3–11 and IGHV3–13 were treated as the same family IGHV3.

### Transcriptional subtypes

Information about the expression-based classification of the samples in the general cohort was obtained from Ref. [40]. Only 184 patients in the general cohort had their expression-based subtype annotated, with 65 of them belonging to the proximal inflammatory (PI) subtype, 51 - to the proximal proliferative (PP) subtype, and 68 - to the terminal respiratory unit (TRU) subtype.

### IgG1 and IgA clonality

To obtain clonality data, we have downloaded the BAM files with reads aligned by STAR from the GDC portal, using the Genomic Data Commons Bioconductor R package (https://bioconductor.org/packages/release/bioc/html/GenomicDataCommons.html). BAM files were then sorted with samtools [41] and converted to Fastq files using the SamToFastq Picard tool (http://broadinstitute.github.io/picard/). MiXCR software [42] was used to extract CDR3 repertoires from Fastq files, and VDJtools [43] was used for the repertoire statistical analysis. Only samples that had more than 500 IgG1 or IgA CDR3-covering sequencing reads were included in the analysis. IgG1 and IgA CDR3 repertoires were downsampled to 500 randomly chosen reads for normalization purposes. Clonality was calculated as: 1 - normalized Shannon-Wiener index [44].

### Survival plots

Survival plots were created using the Kaplan-Meier estimator. Plots were created using matplotlib [45] based on modified functions from the lifelines package (https://zenodo.org/record/2638135#.XMCtiegzaUl). We used a statistical significance threshold of *p* = 0.05. Data analysis was performed with Python2 and R. Multivariable analysis was performed with Cox proportional hazard regression.

### Non-silent mutation burden

The non-silent mutation burden per megabase for each sample was obtained from Ref. [40]. The correlation between IGHG1/IGH proportion and non-silent mutation burden was calculated using Spearman rank correlation coefficient and visualized with Seaborn.

## Results

### *IGHG1/*IGH proportion

**Fig. 1:**
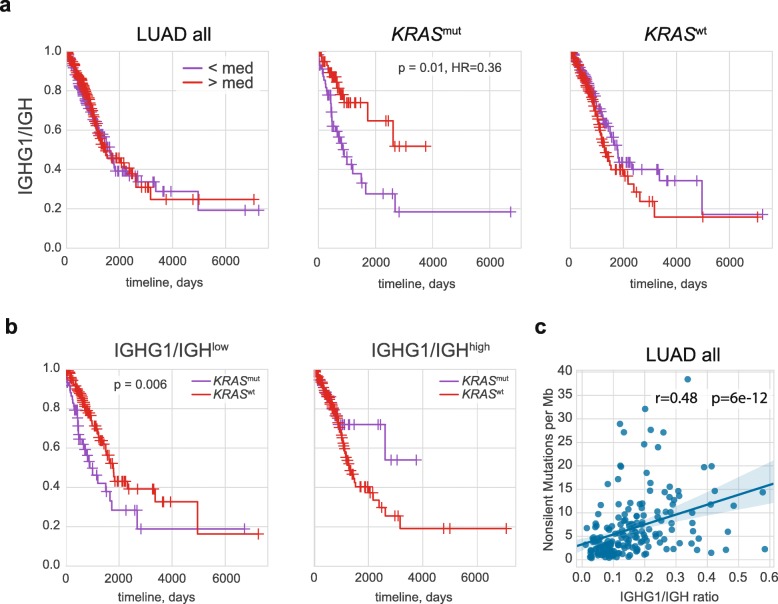
Role of IgG1 expression in LUAD prognosis. **a** Kaplan–Meier overall survival plots for all LUAD patients and patients with *KRAS*^mut^ and *KRAS*^wt^ tumor subtypes are shown as a function of *IGHG1*/IGH ratio, reflecting the proportion of IgG1 of all intratumorally produced antibodies. **b** Kaplan–Meier overall survival plots for patients with low and high *IGHG1*/IGH ratios are shown as a function of *KRAS* status. **c**. Non-silent mutation burden is positively correlated with the IGHG1/IGH ratio

Extrapolating from our previous results obtained with TCGA data for human melanoma [39], we expected to observe an association between a high proportion of *IGHG1/*IGH and long survival, where IGH is a sum of the expression of the *IGHA1, IGHA2, IGHG1, IGHG2, IGHG3, IGHG4, IGHM, IGHD* and *IGHE* genes. However, this was not the case for the TCGA LUAD cohort as a whole (Fig. 1a, hereinafter patient cohorts are split by median).

A broader analysis of each of the cancer types available in TCGA revealed that a high *IGHG1* proportion is only associated with a significantly better prognosis for the full patient cohorts in non-papillary bladder cancer (Bonferroni adjusted *p* < 0.002, HR = 0.5) and melanoma (adjusted *p* < 0.02, HR = 0.6).

However, each cancer type is heterogeneous. It is expected that donors having the same cancer type can show huge variance in survival and immune response owing to the heterogeneity of mutation and gene expression profiles defining cancer phenotype. In an effort to reveal the distinct B-cell characteristics associated with specific types of LUAD and control for potential phenotypic differences, we split patients into 12 partially-overlapping genetic and phenotypic subgroups. These included 3 oncogenic driver status subgroups (*KRAS*^mut^, *KRAS*^wt^, *EGFR*^mut^), 4 tumor suppressor genes subgroups (*p53*^mut^, *p53*^wt^, *STK11*^mut^*, STK11*^wt^), PD-L1^high^ and PD-L1^low^ subgroups, and proximal inflammatory, proximal proliferative, and terminal respiratory unit transcriptional subtypes [40].

Remarkably, out of the 12 subgroups we investigated here, only *KRAS*^mut^ subgroup demonstrated a significant association of a high *IGHG1*/IGH proportion with overall survival (Fig. 1a, adjusted *p* = 0.01, HR = 0.36). A multivariable analysis using Cox proportional-hazards regression with adjustment for stage, gender, smoking, age, and infiltration (CD45 expression) confirmed that a high *IGHG1*/IGH proportion is associated with overall survival for the *KRAS*^mut^ subgroup (p = 0.01, HR = 0.38). In contrast, we observed an inverse - albeit not statistically significant - association in patients with *KRAS*^wt^ tumors (Fig. 1a).

In the *IGHG1*/IGH^low^ subgroup, patients with *KRAS*^mut^ tumor status had a worse prognosis compared to *KRAS*^wt^ (adjusted *p* = 0.006), while in the *IGHG1*/IGH^high^ subgroup, there was no significant difference (Fig. 1b). These results suggest that a high *IGHG1*/IGH proportion plays a protective role in the *KRAS*^mut^ but not in the *KRAS*^wt^ context.

At the same time, in contrast to melanoma, high *IGHG1*/*MS4A1* ratio, reflecting the relative abundance of IgG1-producing plasma cells compared to CD20^+^ (i.e., non-plasma) B-cells, is not associated with longer survival in *KRAS*^mut^ tumors, and tends to have a negative association for *KRAS*^wt^ tumors (data not shown). This observation argues against IgG1-mediated ADCC playing a major protective role in *KRAS*^mut^ LUAD.

Remarkably, the *IGHG1*/IGH proportion is positively correlated with the non-silent mutation burden both for the general LUAD cohort (R = 0.48, *p* = 6 × 10^− 12^, Fig. 1c) and for most LUAD subgroups (Additional file 1: Figure S1). This finding may support the point that IgG1 B cells are involved in the antigen presentation process.

### B-cells versus plasma cells

Abundance of TIBs measured based on *CD19* expression level was associated with a positive prognosis in general LUAD cohort (adjusted *p* = 0.03) and in most subgroups, in agreement with previous works based on immunohistochemical analysis [1, 46], tissue microarrays [47, 48], RNA-Seq [31, 32], and RNA expression microarrays [30, 49].

**Fig. 2:**
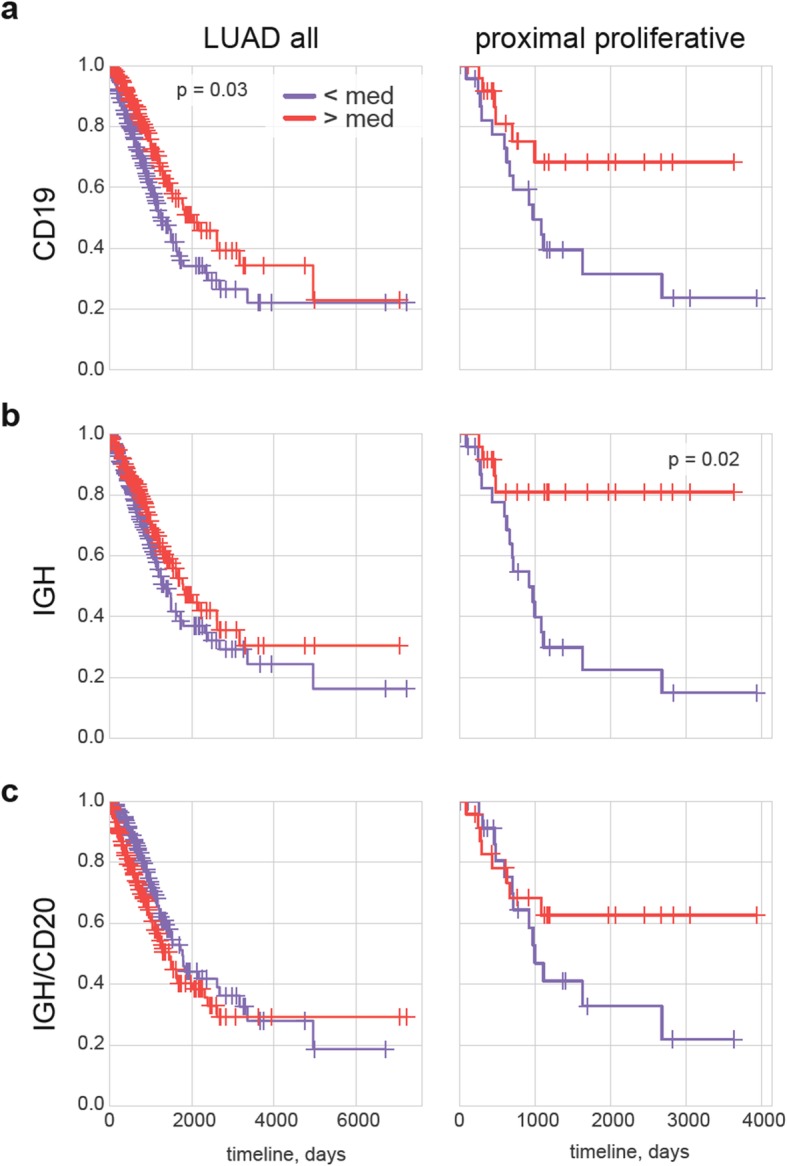
Role of B-cells and antibody-producing plasma cells in LUAD. **a-c** Kaplan–Meier overall survival plots for all LUAD patients as well as patients with the proximal proliferative disease subtype. Survival is plotted as a function of CD19 expression (all B cells, a), IGH expression (antibody production intensity, b) and IGH*/MS4A1* ratio (intensity of antibody production relative to abundance of non-plasma CD20^+^ B cells, c)

However, all of these prior studies have considered LUAD as a general cohort, while here our goal is to find distinct dependencies in LUAD subgroups that can potentially be characterized by different types of the balance in tumor-immunity interactions. Analysis of the 12 subgroups described above has revealed that B-cell infiltration as measured by *CD19* expression level has an especially beneficial impact on survival for the proximal proliferative LUAD transcriptional subtype (Fig. 2a).

Although high IGH expression did not have any meaningful association with survival for most subgroups, it was significantly beneficial for proximal proliferative LUAD (adjusted *p* = 0.02, Fig. 2b). A multivariable analysis using Cox proportional-hazards regression with adjustment for stage, gender, smoking, age, and infiltration confirmed that high IGH expression is associated with prolonged overall survival in proximal proliferative LUAD (*p* = 0.006, HR = 0.08).

High *SDC1* (encoding CD138, indicator of plasma cells) expression was associated with a neutral or non-significant negative effect on overall survival in all cohorts with the exception of proximal proliferative LUAD, where non-significant association with longer survival was observed (data not shown).

We also assessed the ratio of IGH to *MS4A1* (encoding CD20) expression, which reflects the relative abundance of CD20-negative antibody-producing plasma cells compared to CD20-positive non-plasma B-cells. This ratio generally had a slightly negative or neutral effect in all groups, but we observed a non-significant association with positive prognosis in the proximal proliferative LUAD subgroup (Fig. 2c).

We concluded that, although tumor infiltration with CD19-positive B cells is generally a positive signature for most types of LUAD, the presence of antibody-producing plasma cells is specifically associated with better tumor immunosurveillance in the proximal proliferative LUAD subgroup.

### IgG1 antibody production and clonality

We [39] and others [4, 50] have earlier identified an association of high IGH (mainly IgG1 [39]) “clonality” with better survival in melanoma patients, where this metric is calculated as [1 – the normalized Shannon-Wiener index] [44]. In the T-cell world, this metric reflects the relative presence of large clonal expansions. For B-cells, this also reflects RNA expression levels that may differ dramatically between B-cells of differing functional status—with average expression varying by factors of as much as 2:5:500 for naïve, memory and plasma cells, respectively, according to our recent estimations [51]. For highly-infiltrated tumors, antibody CDR3 regions are covered by a relatively large proportion of RNA-Seq reads, which makes it possible to efficiently extract intratumorally-produced immunoglobulin repertoires with MiXCR [39] and thereby analyze clonality metrics. We extracted IgG1 CDR3 repertoires from all patient samples from general LUAD cohort, but only used data from 283 out of 442 patients with > 500 sequencing reads covering IgG1 CDR3, as this is the minimum coverage that allows us to accurately assess clonality [39]. Therefore, this analysis was performed only for tumor samples with relatively high IgG1 expression. For normalization, each dataset was down-sampled to 500 randomly-chosen CDR3-covering sequencing reads.

Notably, high IgG1 clonality, which reflects the presence of a focused IgG1 plasma cell response, did not influence the prognosis for *KRAS*^mut^ LUAD patients (Additional file 1: Figure S2). The neutral effect of both *IGHG1/MS4A1* ratio and IgG1 clonality in *KRAS*^mut^ tumors suggests that IgG1-producing plasma B-cells do not play a prominent role as key drivers of anti-tumor response via ADCC in this subtype of LUAD. In contrast, there is evidence for such a model in melanoma, based on the correlation of large hypermutating clonal IgG1 expansions [39] and high *IGHG1*/*MS4A1* ratio with survival (*p* = 0.006, HR = 0.7), and cytotoxic activity of tumor-specific IgG1 antibodies [8].

One possible interpretation is that in *KRAS*^mut^ tumors, the abundance and high proportion of IgG1 TIBs may play an active positive role via presentation of cognate antigens. Among other TAA and neoantigens, the IgG1 shift of TIBs could lead to more efficient presentation of the mutated KRAS peptide itself. Recent work by the Rosenberg group revealed *KRAS*^mut^-specific CD4^+^ T-cells [52], and *KRAS*-specific tumor-infiltrating IgG B-cells were identified in patients with pancreatic cancer [53]. Ability of lung tumor-infiltrating B-cells to present antigens and activate CD4^+^ T-cells was also reported [18].

Given the fact that a high *IGHG1/*/IGH proportion is associated with longer survival in *KRAS*^mut^, but not *KRAS*^wt^ LUAD cases, we have explicitly tested the repertoires for the presence of specific IgG1 sequence motifs that can be associated with survival. Analysis of IGHV-IGHJ profiles that are most variable across LUAD samples (see **Methods** section) has revealed the presence of a specific signature that is up-regulated in *KRAS*^mut^ cases with high survival (Additional file 1: Figure S3), which is characterized by high abundance of IGHV6-IGHJ4 and IGHV4-IGHJ3 clonotypes and low abundance of IGHV3-IGHJ1 and IGHV3-IGHJ2 clonotypes. These results may indicate that the response to particular tumor antigens is associated with tumor immunosurveillance in *KRAS*^mut^ LUAD, a hypothesis that will require further investigation to confirm.

### IgA and patient survival

High IgA expression levels (the sum of *IGHA1* and *IGHA2* genes) was a neutral parameter in all LUAD subgroups, including *KRAS*^mut^ (data not shown). However, high IgA/IGH (reflecting the proportion of IgA among all intratumorally-produced antibodies) and IgA/*MS4A1* (reflecting IgA production relative to non-plasma B cell abundance) proportions were associated with shorter survival in *KRAS*^mut^ but not in *KRAS*^wt^ patients (Additional file 1: Figure S4a). This dominant presence of IgA could be an indirect consequence of a deficiency in the relative proportion of IgG1 (Fig. 1) and IgG4 (see below), thus leading to a negative prognosis in the *KRAS*^mut^ subgroup. At the same time, the effect of the *IGHG1/MS4A1* parameter was neutral, while increased IgA/*MS4A1* had a negative effect on survival in the *KRAS*^mut^ subgroup (Additional file 1: Figure S4b). This observation supports the negative role of IgA-producing plasma cells in *KRAS*^mut^ LUAD, as was previously reported for hepatocellular carcinoma [33] and bladder cancer [54]. Notably, the IgA clonality parameter remained neutral in all 12 analyzed subgroups (data not shown), suggesting that antigenic specificity of the antibodies produced by the IgA plasma cells does not play a critical role in survival. At the same time, IgA-positive B cells could be involved in antigen presentation, skewing CD4^+^ T cells towards functional phenotypes that are negative or suboptimal for anti-tumor response [18].

### IgG4 expression and patient survival

In 2013, Fujimoto and coauthors used immunohistochemistry analysis to show that the presence of stromal B-cells producing high levels of IgG4 is associated with prominently better prognosis in patients with stage I squamous cell carcinoma [55]. Our analysis extends this observation to LUAD, and delineates a group of LUAD patients that benefit in particular from the presence of IgG4-producing B cells.

**Fig. 3:**
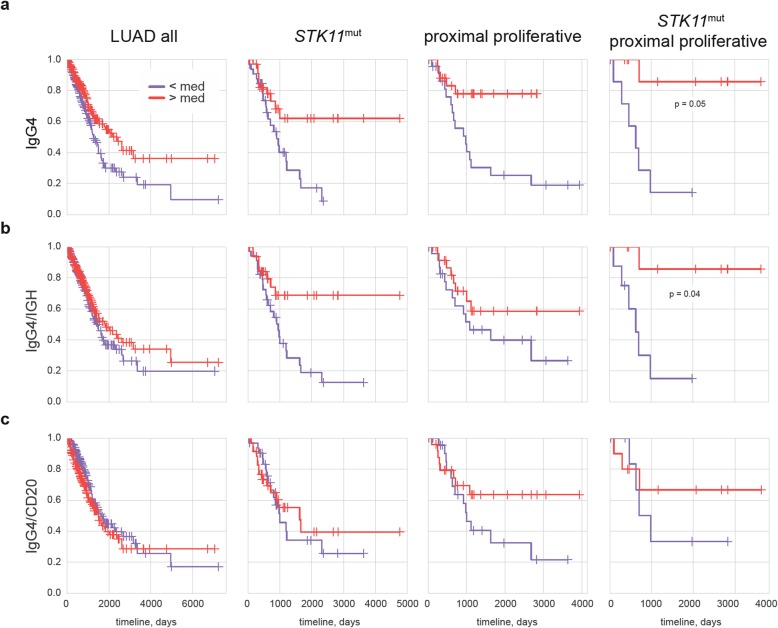
Role of *IGHG4* expression in LUAD. **a-c** Kaplan–Meier survival plots for all patients, *STK11*^mut^, proximal proliferative, and *STK11*^mut^ proximal proliferative LUAD are shown as a function of *IGHG4* expression level (a), *IGHG4*/IGH proportion (IgG4 proportion out of all intratumorally-produced antibodies, b), and *IGHG4*/*MS4A1* ratio (intensity of IgG4 production relative to non-plasma B-cell abundance, c)

High intra-tumoral *IGHG4* expression levels were associated with a better prognosis for the general LUAD cohort (adjusted *p* = 0.06, HR = 0.64). This positive association was based on the positive effect of elevated *IGHG4* levels in *TP53*^wt^ (adjusted *p* = 0.04, HR = 0.49), PD-L1^low^ (HR = 0.64), *STK11*^mut^ (HR = 0.4), and proximal proliferative (HR = 0.26) LUAD (Fig. 3a), whereas no such beneficial effect was observed in other patient subgroups (not shown).

For proximal proliferative LUAD patients, we also found that the overall abundance of several other antibody isotypes, including IgM, IgG1, IgG2, IgG3 - and unexpectedly, IgE - was also associated with better prognosis (Additional file 1: Fig. S5a). This is in agreement with the beneficial role of CD19^+^ B-cells and high IGH expression in this subgroup (Fig. 2a,b). But in terms of relative proportion among all IGH, only high representation of IgG4 – and to some extent IgM, but not other isotypes – tended to be associated with favorable prognosis (Fig. 3b, Additional file 1: Figure S5b).

A high *IGHG4*/IGH proportion was also beneficial for the *STK11*^mut^ subgroup (Fig. 3a,b). A multivariable analysis using Cox proportional-hazards regression with adjustment for stage, gender, smoking, age, and infiltration showed that a high *IGHG4*/IGH ratio was significantly associated with overall survival in *STK11*^mut^ LUAD (*p* = 0.04, HR = 0.4).

Notably, proximal proliferative transcriptional portrait of LUAD tumors is associated with alteration of *STK11* tumor suppressor gene [40], while *STK11* alteration and absence of *TP53* mutations are correlated with PD-L1^low^ LUAD subtype [56, 57]. The patterns that we have observed here thus could represent components of the same picture, describing the cumulative tumor portrait of lung adenocarcinoma patients that mostly benefit from abundant intratumoral IgG4 expression as *TP53*^wt^/*STK11*^mut^/PD-L1^low^/proximal proliferative LUAD.

Indeed, for the small subgroup of 19 patients with both *STK11*^mut^ and proximal proliferative LUAD, the positive association of survival with high *IGHG4* expression (adjusted *p* = 0.05) and proportion (adjusted p = 0.04) was even more prominent (Fig. 3).

When we normalized to CD20—measuring relative IgG4 production compared to the abundance of non-plasma B-cells—we determined that a high *IGHG4/MS4A1* ratio only tended to be associated with a positive prognosis in proximal proliferative LUAD (Fig. 3c).

## Discussion

Clearly, the impact of B-cells in cancer immunology is not black and white, and we cannot simply distinguish “positive” IgG1/IgG3 isotypes that initiate tumor-specific ADCC and immune responses from “negative” IgA/IgG4 isotypes as a signature of or precursor to immunosuppression. The particular antigenic specificities of intratumoral BCRs/antibodies - which include the surface versus intracellular localization of cognate tumor antigens - and associated phenotypes of antigen-presenting and cytokine-producing B-cells all contribute to the complex picture of tumor-immunity interactions.

Nevertheless, here we have delineated LUAD patient subgroups that can be characterized by striking dependencies between the abundance and proportion of particular intratumorally-produced BCR/antibody isotypes and survival. Association of IgG1 and IgG4 isotype dominance with favorable prognosis in *KRAS*^mut^ and *STK11*^mut^/proximal proliferative LUAD patients, respectively, hints at the existence of specific types of established tumor-immunity interaction profiles. The latter could either involve driver mutations themselves in antigen-specific response [52, 53, 58] or result from downstream pathways characteristic for specific driver mutation.

This discovery of existing links between driver mutations and TIB-mediated immunity complements recently described interconnections between driver mutations, T-cell behavior, and PD-L1 expression [56, 59]. In particular, the *KRAS*^G12D^ mutation and MEK/ERK pathway activation was shown to up-regulate production of IL-10 and TGF-β, thus promoting CD4 T cells conversion in Tregs in pancreatic cancer [60]. In LUAD, *KRAS* mutation was associated with more intense immune cells infiltration [57]. Several other driver mutations correlated with lower or higher leukocyte infiltration across all cancer types [61].

The positive influence of IgG1 TIBs in *KRAS*^mut^ tumors could be explained by presentation of BCR-cognate tumor antigens to CD4^+^ T-cells. Considered alongside recent reports revealing the importance of antigen-specific B-cells as cognate antigen presenters [6, 7, 18, 62, 63], these results support the concept that therapeutic vaccination using whole proteins or their encoding genes (including *KRAS*^mut^) [64] could more efficiently exploit the antigen-presentation machinery of cognate B-cells. The hypothesis that mutant KRAS peptide itself is among the involved antigenic targets is especially attractive since, in contrast to other neoantigens, the driver mutation is a sensitive component of tumor survival. However, exploring this hypothesis in depth will require further investigation.

The reason for the observed association of high IgG4 production with a favorable prognosis in *STK11*^mut^ and proximal proliferative LUAD remains unclear, and will require further progress in our fundamental understanding of the functionality of the IgG4 isotype.

We hypothesize the following explanation. In IgG4, inter-heavy chain disulfides are in equilibrium with intra-heavy chain disulfides [65], which enables heavy chain monomer exchange in vivo [66]. As a result, IgG4 functions as a monovalent antibody, which is unable to cross-link antigen and form immune complexes [66]. Notably, persistent immune complexes formed by tumor-specific antibodies may be associated with an unfavorable clinical outcome [67] due to their immunosuppressive action through the modulation of FcR-bearing myeloid cell activity, leading to a MDSC phenotype [23, 24]. Thus, a positive role of IgG4 in lung cancer could be connected with diminished formation of immune complexes and subsequent MDSC-related immunosuppressive reactions. At the same time, it should be noted that antibodies of IgG4 isotype may also have a negative impact on prognosis for some cancer types, as has been reported for human melanoma [68].

## Conclusion

Our discovery of direct links existing between antibody isotypes and survival in lung adenocarcinoma carrying specific driver mutations strengthens the importance of TIBs as immune system players with multi-parametric roles in the battle with cancer. This may suggest prospective strategies for more rational design of combination approaches incorporating targeted therapies, immune checkpoint inhibitors, and vaccines. In particular, these results indicate that immunotherapy efforts must take into account B-cell component of the tumor microenvironment, which role, most importantly, may depend on particular context of driver mutations.

## Supplementary information

**Additional file 1: Figure S1.** Non-silent mutation burden correlates with IGHG1/IGH proportion in LUAD subgroups. **Figure S2.** IgG1 clonality. **Figure S3.** Exploring IGH motifs linked to IgG1-mediated survival in *KRAS*^mut^ LUAD. **Figure S4.** Role of IgA expression in LUAD. **Figure S5.** Immunoglobulin isotypes and proportions in proximal proliferative LUAD. (DOCX 1198 kb)

## Data Availability

Extracted IGH, IgA, and IgG1 CDR3 repertoires, metadata, expression and clonality metrics are deposited on Figshare: https://figshare.com/projects/BCR_profiling_in_lung_adenocarcinoma_TCGA_cohort/64106.

## Abbreviations

ADCC: Antibody-Dependent Cellular Cytotoxicity
BCR: B-cell receptor
FFPE: Formalin-Fixed Paraffin-Embedded tissue
FPKM: Fragments Per Kilobase Million
LUAD: LUng ADenocarcinoma
MDSC: Myeloid-Derived Suppressor Cell
TAA: Tumor-Associated Antigens
TCGA: The Cancer Genome Atlas
TIBs: Tumor-Infiltrating B-cells
