# Supplementary material for: Intratumoral immunoglobulin isotypes predict survival in lung adenocarcinoma subtypes
Source: J Immunother Cancer. 2019 Oct 29;7:279. doi: 10.1186/s40425-019-0747-1 (PMC6819482; doi:10.1186/s40425-019-0747-1)
Supplement: Supplementary file 1 — Additional file 1: Figure S1. Non-silent mutation burden correlates with IGHG1/IGH proportion in LUAD subgroups. Figure S2. IgG1 clonality. Figure S3. Exploring IGH motifs linked to IgG1-mediated survival in KRASmut LUAD. Figure S4. Role of IgA expression in LUAD. Figure S5. Immunoglobulin isotypes and proportions in proximal proliferative LUAD. (DOCX 1198 kb) [file 40425_2019_747_MOESM1_ESM.docx]

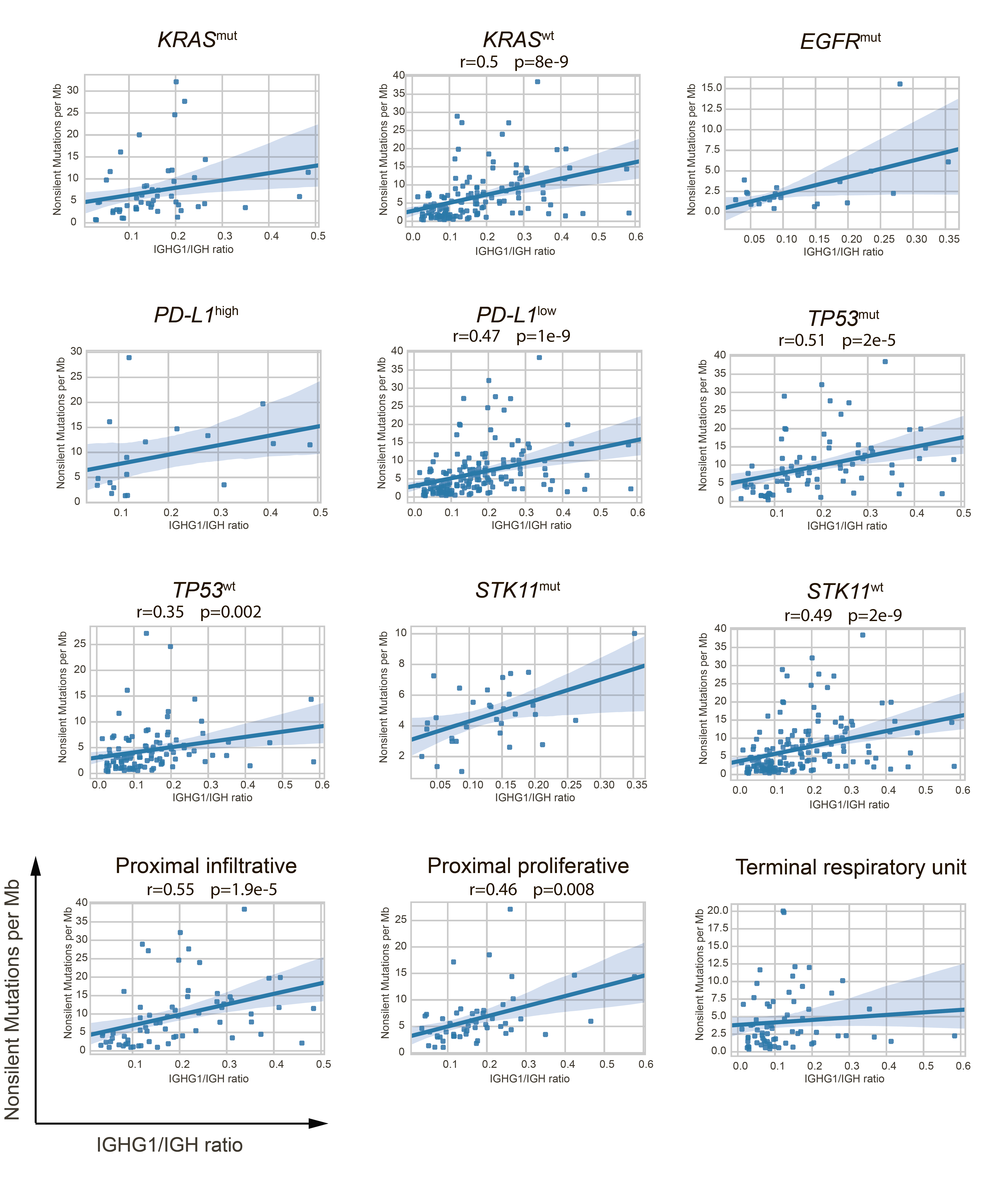


**Figure S1. Non*-*silent mutation burden correlates with IGHG1/IGH proportion in LUAD subgroups.** Spearman rank correlation, Bonferroni adjusted *p* values are shown.


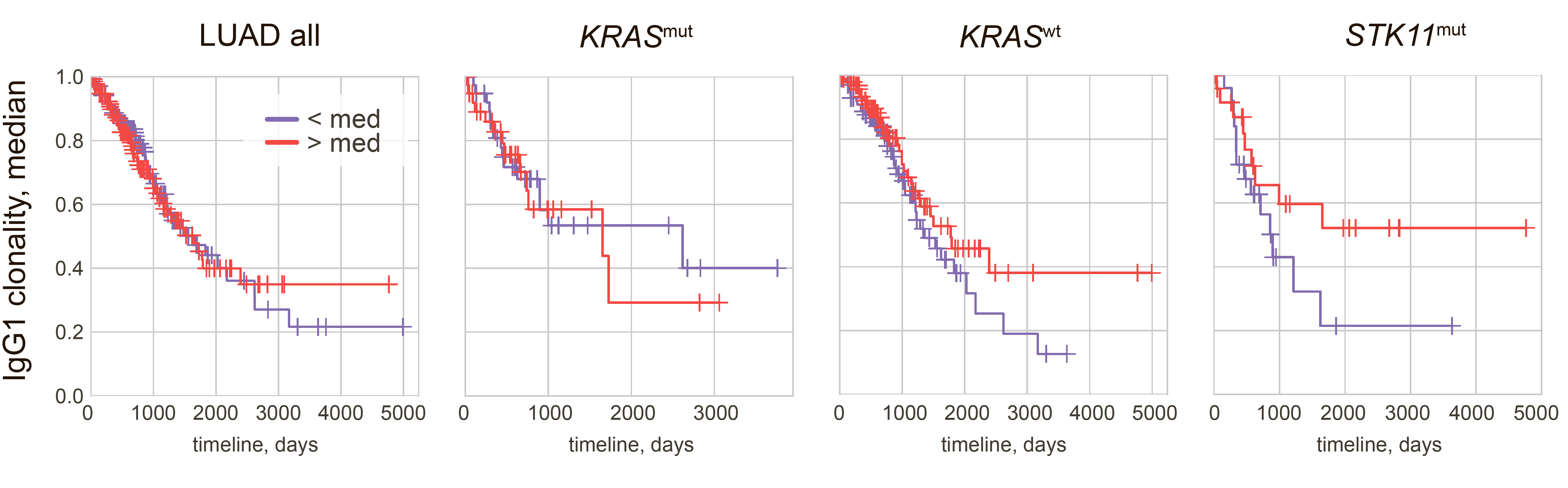


**Figure S2. IgG1 clonality.** Kaplan–Meier overall survival plots for all LUAD patients, *KRAS*^mut^, *KRAS*^wt^, and *STK11*^mut^ cases as a function of IgG1 clonality (1 – the normalized Shannon-Wiener index).


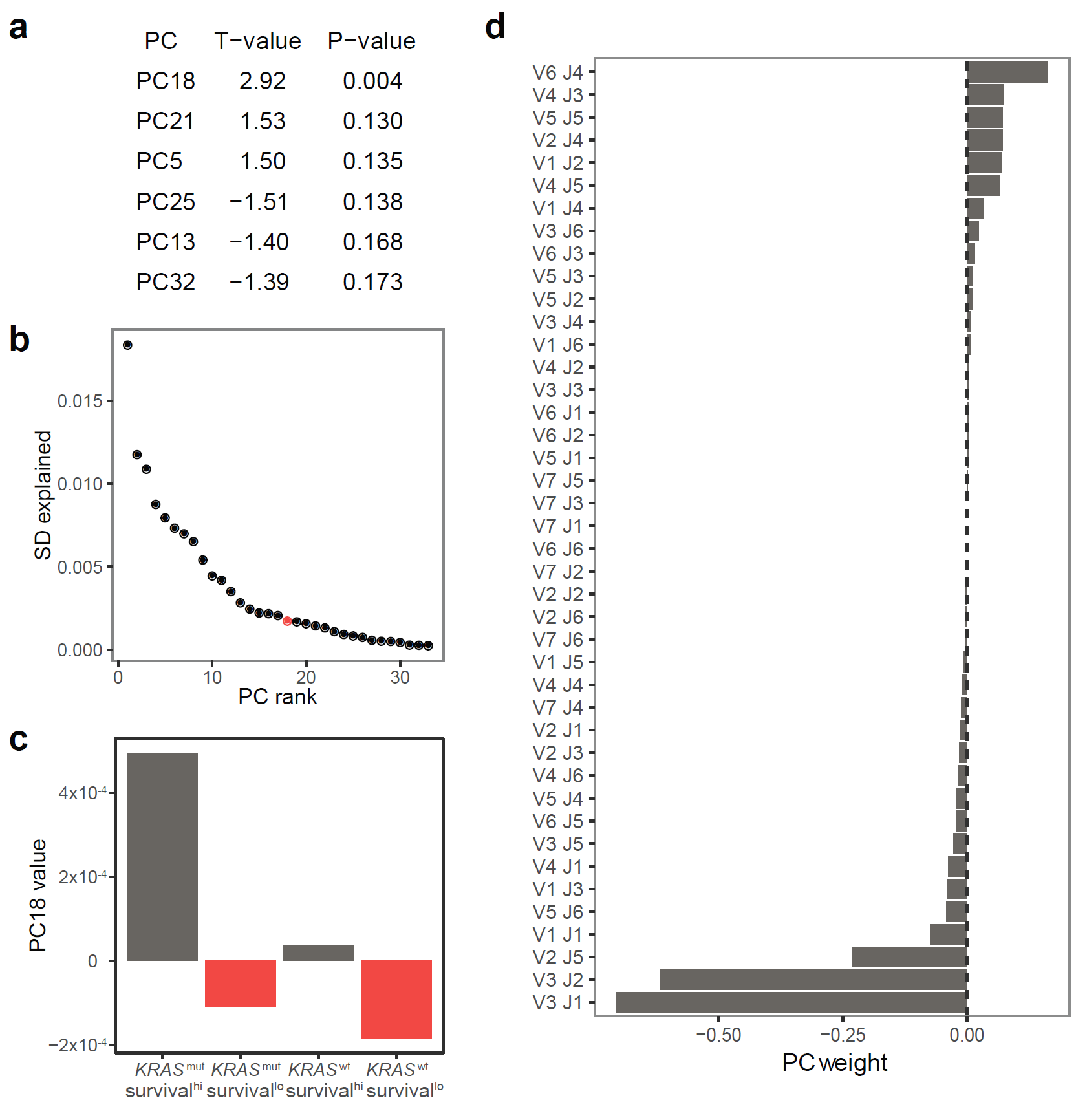


**Figure S3. Exploring IGH motifs linked to IgG1-mediated survival in *KRAS*^mut^ LUAD. a.**Top 6 principal components (PCs) of the IGHV-IGHJ profile across by their T-score (two-tailed T-test) for expression in *KRAS*^mut^ high-survival versus other LUAD samples. **b.** Variance explained by PCs of IGHV-IGHJ profile in all LUAD samples. Component having P < 0.05 is highlighted with red point. **c**. Mean value of selected PC18 component in *KRAS*^mut/wt^ high and low survival cases. **d**. Scores for IGHV-IGHJ combinations that contribute to the PC18 component.


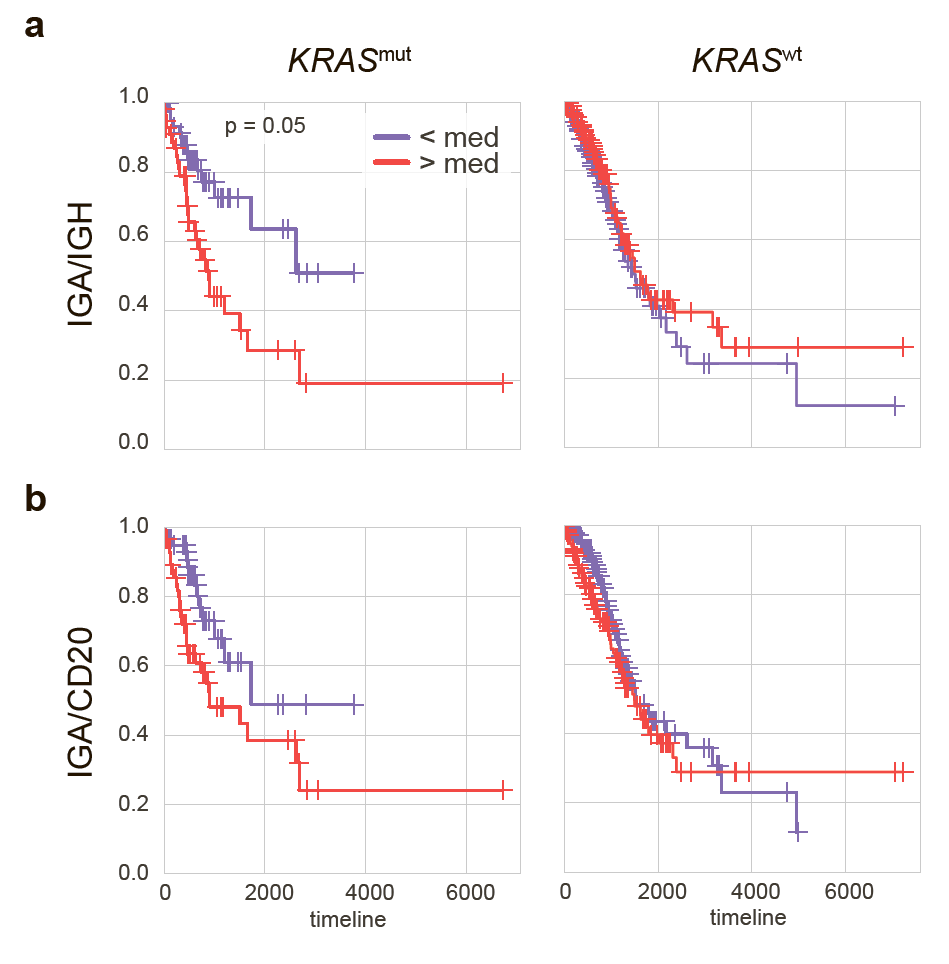


**Figure S4**. **Role of IgA expression in LUAD. a**,**b**. Kaplan–Meier overall survival plots for *KRAS*^mut^ and *KRAS*^wt^ patients are shown as a function of IgA/IGH proportion (IgA proportion out of all intratumorally produced antibodies, a), and IgA/*MS4A1* ratio (intensity of IgA production relative to non-plasma B cell abundance, b) expression level ratios.


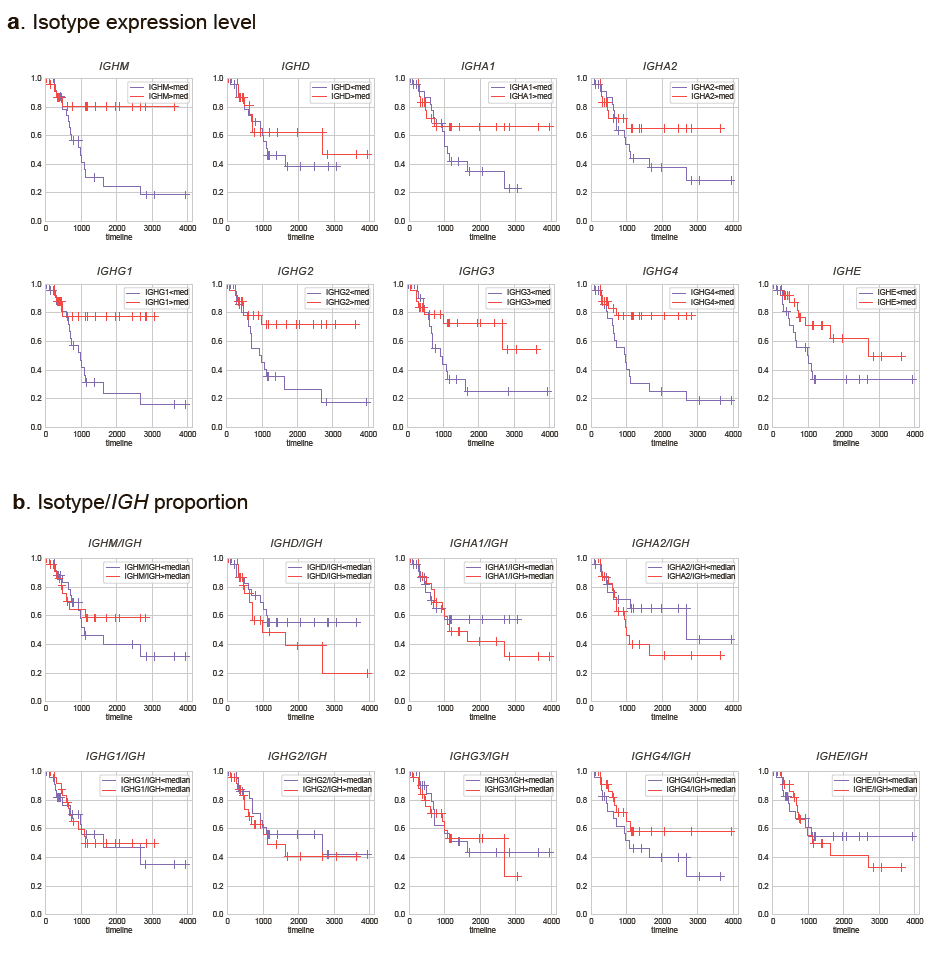


**Figure S5.** **Immunoglobulin isotypes and proportions in** **proximal proliferative LUAD. a**,**b**. Kaplan–Meier overall survival plots are shown as a function of immunoglobulin isotype expression level (a) and isotype/IGH ratio (isotype proportion out of all intratumorally produced antibodies, b).
